# Sleep-Related Predictors of Risk for Alcohol Use and Related Problems in Adolescents and Young Adults

**DOI:** 10.35946/arcr.v44.1.02

**Published:** 2024-03-14

**Authors:** Brant P. Hasler, Christina T. Schulz, Sarah L. Pedersen

**Affiliations:** Department of Psychiatry, University of Pittsburgh School of Medicine, Pittsburgh, Pennsylvania

**Keywords:** alcohol, adolescent, sleep, circadian rhythm, young adult, experimental model, longitudinal studies, research design

## Abstract

**PURPOSE:**

Growing evidence supports sleep and circadian rhythms as influencing alcohol use and the course of alcohol use disorder (AUD). Studying sleep/circadian–alcohol associations during adolescence and young adulthood may be valuable for identifying sleep/circadian-related approaches to preventing and/or treating AUD. This paper reviews current evidence for prospective associations between sleep/circadian factors and alcohol involvement during adolescence and young adulthood with an emphasis on the effects of sleep/circadian factors on alcohol use.

**SEARCH METHODS:**

The authors conducted a literature search in PsycInfo, PubMed, and Web of Science using the search terms “sleep” and “alcohol” paired with “adolescent” or “adolescence” or “young adult” or “emerging adult,” focusing on the title/abstract fields, and restricting to English-language articles. Next, the search was narrowed to articles with a prospective/longitudinal or experimental design, a sleep-related measure as a predictor, an alcohol-related measure as an outcome, and confirming a primarily adolescent and/or young adult sample. This step was completed by a joint review of candidate article abstracts by two of the authors.

**SEARCH RESULTS:**

The initial search resulted in 720 articles. After review of the abstracts, the list was narrowed to 27 articles reporting on observational longitudinal studies and three articles reporting on intervention trials. Noted for potential inclusion were 35 additional articles that reported on studies with alcohol-related predictors and sleep-related outcomes, and/or reported on candidate moderators or mediators of sleep–alcohol associations. Additional articles were identified via review of relevant article reference lists and prior exposure based on the authors’ previous work in this area.

**DISCUSSION AND CONCLUSIONS:**

Overall, the review supports a range of sleep/circadian characteristics during adolescence and young adulthood predicting the development of alcohol use and/or alcohol-related problems. Although sleep treatment studies in adolescents and young adults engaging in regular and/or heavy drinking show that sleep can be improved in those individuals, as well as potentially reducing alcohol craving and alcohol-related consequences, no studies in any age group have yet demonstrated that improving sleep reduces drinking behavior. Notable limitations include relatively few longitudinal studies and only two experimental studies, insufficient consideration of different assessment timescales (e.g., day-to-day vs. years), insufficient consideration of the multidimensional nature of sleep, a paucity of objective measures of sleep and circadian rhythms, and insufficient consideration of how demographic variables may influence sleep/circadian–alcohol associations. Examining such moderators, particularly those related to minoritized identities, as well as further investigation of putative mechanistic pathways linking sleep/circadian characteristics to alcohol outcomes, are important next steps.

Abundant cross-sectional data indicate that alcohol use and related problems are accompanied by disruptions to sleep and circadian rhythms.^1^ Alcohol’s negative impacts on sleep are well established, especially in adults, and a smaller body of literature also reports alcohol’s disruption of circadian rhythms.^2^–^4^ Growing evidence supports sleep and circadian factors as influencing alcohol use and related problems, including as risk factors for the initial development of use and problems, as predictors for relapse in individuals with alcohol use disorder (AUD), and as targets for intervention.^2^,^5^–^7^ Given the marked changes in sleep and circadian rhythms that occur throughout adolescence into young adulthood,^5^ paralleling the time frame when initial alcohol use and development of alcohol-related problems are most likely to occur,^8^ there may be particular value to studying the association between sleep/circadian rhythms and alcohol during this developmental stage.

## Sleep and Circadian Changes in Adolescence and Beyond

As a result of living on a rotating planet with alternating light and dark periods, humans and most other living organisms have evolved to experience internal biological rhythms lasting approximately 24 hours.^9^ These circadian rhythms modulate the timing of many, if not most, physiological, behavioral, and psychological processes, including the sleep-wake cycle, with the goal of optimizing temporal relationships with the environment and with one another. Notably, the timing of circadian rhythms is not static but shows developmental changes. Starting with the onset of puberty, the timing of sleep and circadian rhythms shifts later throughout adolescence, peaking around age 20 before reversing course and slowly shifting earlier over the rest of the life span.^10^,^11^ The changes in sleep timing are driven by both biological and sociocultural factors and thus can vary based cross-nationally^12^ and on sociodemographic characteristics.^11^,^13^ Biological factors include the changes in circadian rhythms as well as changes in homeostatic sleep propensity, which accumulates more slowly during adolescence.^14^ Exposure to blue light (e.g., via electronic devices) in the evening can exacerbate these tendencies toward later sleep and circadian timing.^15^,^16^

Although the need for sleep remains relatively stable during this period—with recommendations for 8 to 10 hours/night in youth ages 13 to 17 and for 7 to 9 hours/night in people age 18 and older—actual sleep duration tends to decrease, especially on school/work nights.^14^,^17^ This reduction in sleep duration is driven in part by a mismatch between the tendency for later sleep/circadian timing and relatively early school schedules, particularly during middle school and high school. This mismatch, termed circadian misalignment or social jet lag, not only results in insufficient sleep duration, but also can contribute to difficulty falling asleep on school nights, daytime sleepiness on school days, and large swings in sleep timing and duration on weekdays versus weekends.^14^ Such swings tend to manifest as later sleep timing and shorter sleep duration, especially for those with later circadian timing.^18^ Although the effects of early school start times are most systematic during secondary education, circadian misalignment and the associated constellation of sleep problems can persist well after high school. Regardless of etiology, insomnia, insufficient sleep, and social jet lag remain prevalent in the years after high school graduation into people’s twenties,^18^–^20^ although prevalence varies based on sociodemographic characteristics.^21^

Sleep is multifactorial, and as illustrated above, different facets of sleep are interrelated in complex ways.^22^,^23^ Circadian misalignment and social jet lag are often accompanied by a constellation of sleep-related problems and thus cannot be adequately captured by only assessing sleep quality, sleep duration, or sleep timing, especially if not distinguishing between school days or workdays and free days.

## Alcohol Trajectories in Adolescence and Beyond

The developmental span from adolescence to young adulthood is a time of increasing alcohol use and related problems.^8^ Alcohol use then tends to decline in early adulthood as individuals begin to “mature out” due to increases in adult responsibilities.^24^ Further, both earlier initiation of alcohol use and more rapid progression from initiation to intoxication have been found to predict problematic alcohol use later on.^25^–^27^ Multiple explanatory mechanisms are thought to underlie the onset and progression of risky alcohol use in adolescence through early adulthood. In particular, heightened sensation seeking and impulsivity have been consistently identified as potential risk factors for problematic alcohol use^28^–^33^ and are related to sleep and circadian factors.^34^,^35^

## Overview of Alcohol’s Effects on Sleep

The effects of alcohol on sleep and, to a lesser extent, circadian rhythms in adult samples have been thoroughly and recently reviewed,^2^–^4^ so are only briefly discussed here. Given the bidirectional relationships between sleep and alcohol use, a brief summary of the evidence for alcohol’s effects on sleep and circadian rhythms is warranted as it provides important context in interpreting observational data where it is impossible to fully parse these bidirectional effects.

Alcohol administration studies in adults have assessed alcohol’s acute effects on sleep via polysomnography, which measures brain activity (electroencephalography [EEG]), eye movements, muscle activity, and cardiac activity. These studies found that during the first half of the night, alcohol tends to shorten the time it takes to fall asleep (sleep onset latency [SOL]), reduce nighttime wakefulness (i.e., decrease wake after sleep onset [WASO]), decrease rapid eye movement (REM) sleep, and increase the deepest of the non-REM sleep stages (i.e., slow-wave sleep).^2^ (See Box: Glossary of Sleep-related Terms for more detailed definitions.) However, during the second half of the night, alcohol tends to acutely increase WASO and reduce sleep efficiency (the percentage of time spent asleep relative to the time spent attempting to sleep), while leading to a rebound in REM sleep.^2^ Overall, polysomnography studies suggest that adults spend more time awake on nights after consuming alcohol.^2^ Some sex differences in the acute effects of alcohol have been noted, as described below.

**Glossary of Sleep-related Terms**

**Actigraphy:** Noninvasive and objective method of measuring rest-activity patterns, and thereby estimating sleep-wake characteristics, via a wearable device containing an accelerometer. Most typically worn on the wrist.

**Chronotype:** Tendency toward relatively earlier or relatively later timing of the circadian clock, often as indexed by timing of the sleep-wake schedule. Conceptually overlaps with circadian preference and/or morningness-eveningness—the self-reported preference for relatively earlier (morningness) or later (eveningness) patterns of activity and sleep.

**Circadian misalignment:** Mismatch between the timing of the behavioral sleep-wake schedule and that of the circadian clock, most obviously observed in the context of shiftwork and jet lag.

**Eveningness:** Self-reported preference for relatively later timing of sleep and activity. In contrast to morningness, a self-reported preference for relatively earlier timing of sleep and activity. See chronotype.

**Polysomnography:** A multiparameter assessment of sleep that includes electroencephalography (EEG) to assess brain activity, electrooculography (EOG) to assess eye movements, electromyography (EMG) to assess muscle activity, and electrocardiography (ECG) to assess cardiac activity. Often respiratory airflow, respiratory effort, and pulse oximetry are also measured. Typically applied in laboratory-based settings, although streamlined polysomnography-type devices are increasingly used in home settings.

**Sleep efficiency:** The percentage of time spent asleep relative to the time spent attempting to sleep.

**Sleep onset latency (SOL):** The amount of time it takes to fall asleep.

**Slow-wave sleep:** The deepest of three stages of non-rapid eye movement (non-REM) sleep.

**Social jet lag:** A specific type of circadian misalignment in which school and/or work obligations cause a mismatch between the imposed sleep-wake schedule on school days or workdays, whereas individuals return to their desired sleep-wake schedules (relatively more aligned with their circadian clocks) on free days. More common are individuals with a late chronotype (tendency toward evening circadian preference).

**Wake after sleep onset (WASO):** The amount of time spent awake during nighttime awakenings that occur after initially falling asleep.

Acute alcohol effects in adolescents have been much less studied, but findings suggest some distinctions from the effects observed in adults. In a study with 24 participants ages 18 to 21 (12 women) with a mean breath alcohol content of 0.084% at lights out, alcohol’s effects were broadly similar but with less evidence of benefits for sleep. Specifically, adolescents did not exhibit the decrease in SOL or the REM rebound,^36^ and although alcohol appeared to increase delta power (EEG activity in the 1–4 Hz range; typically highest during slow-wave sleep) during the first few sleep cycles, it simultaneously increased alpha power (EEG activity in the 8–13 Hz range; associated with quiet wakefulness) in frontal regions.^37^ This alpha-delta pattern in response to alcohol has been observed in some but not all prior studies^38^,^39^ and is thought to reflect disrupted sleep. No sex differences were reported.

As reviewed by Koob and Colrain,^2^ alcohol’s effects on sleep—when alcohol use is more chronic and/or when people who chronically use alcohol (i.e., patients with AUD) abstain from drinking—can diverge from the acute effects of alcohol in complex ways too nuanced to adequately review here. Generally, chronic alcohol use is associated with worse sleep (e.g., more insomnia, longer SOL and WASO), although sleep may intermittently improve on drinking nights; similarly, abstinence is typically associated with initial worsening of sleep with some incremental improvement over time.^2^ Various sleep abnormalities persist in individuals with AUD, even with long-term abstinence (> 30 days). A recent meta-analysis of cohort studies in broader samples underscores the general conclusion that chronic alcohol use does not improve sleep overall, and likely increases the likelihood of developing sleep disorders over time.^40^

Although intensive longitudinal studies cannot confirm causality or directionality, analyses of day-to-day alcohol–sleep associations in young adults suggested that drinking on a given day was associated with later sleep timing that night.^41^,^42^ Interestingly, such analyses offered mixed evidence for whether drinking worsened^43^ or improved^42^ sleep. Additionally, some studies in young adults have shown that cannabis use may mitigate alcohol’s effects on sleep,^43^,^44^ although these studies require replication, and the relevant mechanisms remain unknown.

Alcohol’s effects on sleep also depend on the timing of alcohol consumption; for example, a study in middle-aged men administered alcohol 6 hours before bedtime found no benefit for SOL.^45^ This likely was due to a combination of the temporal dynamics of the biphasic response to alcohol and circadian variation in the response to alcohol. While the literature on alcohol effects on circadian rhythms is more limited, particularly in humans,^3^,^4^ studies have suggested disruption of melatonin and core body temperature rhythms. Multiple animal studies have indicated that acute and chronic alcohol use disrupted the circadian system’s response to light, which is the most important cue (zeitgeber, or time giver) for entraining to the 24-hour day.^46^,^47^

Although parallel effects in humans were not supported by one study in healthy adults reporting light or regular but not heavy alcohol use,^48^ more recent work suggested reduced retinal responsivity to light in a group of adults who drank heavily.^49^ Light or regular drinking has previously been defined as “consumption of one to five standard alcoholic drinks/week” and no more than three episodes of binge drinking in the past year.^49^ Heavy drinking has been defined by the National Institute on Alcohol Abuse and Alcoholism as five or more drinks on any day or 15 or more drinks per week for men, or four or more drinks on any day or eight or more drinks per week for women (see https://go.nih.gov/TiogZz9). However, there is no standardized definition of either “light/regular drinking” or “heavy drinking” across the studies described in this article.

The present paper reviews current evidence for prospective associations between sleep/circadian factors and alcohol involvement during adolescence and young adulthood, with an emphasis on the effects of sleep/circadian factors on alcohol use and related outcomes. This focus was selected in part because identifying modifiable sleep–alcohol relationships during this developmental period offers the potential for shifting the trajectory for alcohol-related problems before they develop into chronic AUD. This article also describes and discusses potential mechanisms by which sleep may influence alcohol use and problems, as well as potential important differences in sleep–alcohol associations based on key moderators, such as assigned sex at birth; lesbian, gay bisexual, transgender, queer/questioning, intersex, and asexual (LGBTQIA+) identities; and racial and ethnic identities.

## Methods

### Search Methods

The initial search of the existing literature was conducted on July 18, 2022, in PsycInfo, PubMed, and Web of Science using the search terms “sleep” and “alcohol” paired with “adolescent” or “adolescence” or “young adult” or “emerging adult,” in the title or abstract fields; results were restricted to English-language articles but had no restriction by date. Next, the search was narrowed by including only articles that had a prospective/longitudinal or experimental design, included a sleep-related measure as a predictor, assessed an alcohol-related measure as an outcome, and had a sample primarily composed of adolescents and/or young adults. Based on these search terms, the resulting ages of participants in the articles ranged from ages 12 to 30. Table 1 offers information on age ranges in specific studies. Two of the authors completed this step by conducting a joint review of candidate article abstracts.

### Results of the Literature Search

The initial search resulted in 720 articles (174 in PsycInfo, 305 in PubMed, and 241 in Web of Science). After review of the abstracts to identify articles that met all the key search criteria, the list was narrowed to 27 articles reporting on observational longitudinal studies and three articles reporting on experimental studies (specifically, two intervention trials). Noted for potential inclusion were 35 additional articles that reported on studies with alcohol-related predictors and sleep-related outcomes, and/or reported on candidate moderators or mediators of sleep–alcohol associations. An additional 104 articles cited here were identified via a variety of methods, including review of relevant article reference lists and prior exposure based on the authors’ previous work in this area. Finally, while this review focused on sleep/circadian–alcohol associations in human studies, a few select findings from three animal studies^46^,^47^,^50^ were included when they appeared particularly complementary to the human findings and/or helped speak to a gap in the human literature.

## Results of the Reviewed Studies

### Longitudinal Sleep and Alcohol Studies

Overall, the existing literature—based on 27 articles, including three intensive longitudinal studies—provides consistent evidence that a range of sleep/circadian factors during adolescence predicts later alcohol involvement. These included difficulties with falling or staying asleep, lower overall sleep quality, shorter sleep duration, daytime sleepiness, later sleep timing and/or chronotype (i.e., tendency for relatively earlier or later sleep-wake timing), and variable sleep timing and/or social jet lag (see Box: Glossary of Sleep-related Terms). Alcohol-related outcomes assessed included metrics of both quantity and frequency of use, binge or heavy drinking episodes, alcohol intoxication, alcohol-related consequences/problems, AUD symptoms, and alcohol craving. Table 1 provides a summary of the longitudinal studies, including sample composition, study design, and timescale; which multidimensional sleep variables were predictive of alcohol outcomes; and whether differences across assigned sex, gender identity, and racial/ethnic identity were assessed.

A majority of the articles^5^,^6^,^51^–^63^ also reported on other substance outcomes, particularly use of nicotine/tobacco and cannabis/marijuana, with findings suggesting that sleep-related risk for substance use may not be specific to alcohol. Indeed, the overall literature suggests a transdiagnostic scenario where multiple aspects of sleep/circadian disturbance (e.g., insomnia, sleep loss, delayed phase) increase the risk for alcohol and other substance use disorders as well as for other psychiatric disorders.^64^

Although this review focuses primarily on the period of adolescence through young adulthood, two papers based on the Michigan Longitudinal Study^61^,^62^ and one paper based on a study in Hong Kong^65^ reported that childhood sleep problems predicted later substance use, indicating that relationships between sleep and substance use are not specific to adolescents. Notably, childhood sleep tends to predict adolescent sleep,^62^,^65^ which could partially explain the association with adolescent substance use, but also suggests the potential value of starting early with sleep-focused prevention and/or intervention efforts. Indeed, one study reported prospective sleep–substance use associations entirely within the fourth through sixth grades, and implicated inhibitory control as a potential mediator.^66^ Although that study’s findings contrasted with one of the papers from the Michigan Longitudinal Study (which did not support inhibitory control as a mediator in the sleep–alcohol associations),^62^ changes in mood regulation, impulsivity, and/or poor decision-making remain plausible mechanisms in the longitudinal associations between childhood sleep problems and later substance use.

Several caveats are important to consider when interpreting the existing literature. First, multiple articles relied on the same longitudinal datasets; thus, 14 out of 27 longitudinal papers were based on six studies. Second, earlier studies tended to focus on only one or two sleep characteristics and were thus unable to treat sleep as a multidimensional construct. Third, papers based on more recent studies, seemingly designed to specifically consider sleep, were more likely to employ a multidimensional sleep framework.^5^,^6^,^41^,^53^,^67^–^69^ Fourth, except for two intensive longitudinal studies^41^,^42^ that used actigraphy—a wearable device containing an accelerometer to measure rest-activity patterns—most studies relied on self-reported sleep and are subject to the relevant biases. For example, beyond typical retrospective biases associated with self-report, there are also longstanding observations of subjective-objective discrepancies in sleep, particularly in individuals with insomnia disorder.^70^ Also, none of the studies included objective circadian predictors (e.g., dim light melatonin onset) despite cross-sectional evidence that circadian timing is related to alcohol outcomes.^49^,^71^ Fifth, observational designs cannot assess causation and directionality and therefore must be interpreted with caution. Relatedly, one recent co-twin study indicated that sleep-related risk for alcohol misuse exists over and above genetic and environmental factors.^72^ However, other emerging research using genetic methods has yielded more mixed results whether the relationships between sleep/circadian characteristics and substance use should be attributed to shared genetic variance or pleiotropy^73^–^75^ or suggests a causal relationship from sleep to substance misuse.^76^

Some of the included studies tested putative mediators of the sleep–alcohol relationship, such as behavioral inhibition, attention problems, and internalizing/externalizing symptoms; however, the results have been inconsistent (see below for further discussion). Furthermore, given that a tendency toward relatively late timing of the sleep-wake schedule (i.e., a later chronotype) is often associated with worse sleep among adolescents and young adults,^77^ sleep characteristics are a putative mediator of the association between chronotype and alcohol-related risk. However, existing studies often have not supported this for alcohol^78^ or other outcomes such as depression.^79^,^80^ One study in late adolescents and young adult veterans reported that insomnia severity statistically mediated the association between depression or symptoms of post-traumatic stress disorder and alcohol use and related consequences.^81^

The time frame of assessment varied substantively across the studies, with intensive longitudinal designs narrowing the focus to day-to-day relationships whereas the more traditional longitudinal studies ranged from months to multiple years between assessments. These varying time frames are important when considering that distinct mechanistic pathways may be operating within different timescales. For example, studies with annual or multiannual time points may be speaking more to the cumulative effects of sleep/circadian characteristics, although few studies have directly tested this.^65^ Interestingly, the intensive longitudinal designs (e.g., ecological momentary assessment [EMA]) appear more likely to find more nuanced associations between sleep and alcohol. For example, some EMA evidence from young adult samples suggests that better sleep efficiency^42^ or quality^41^ on a given night predicts more alcohol use the following day, although those findings emerged from samples with participants with sleep problems who consume alcohol. EMA findings from a much wider age range (ages 20 to 73) suggest that age may moderate sleep–alcohol associations; the younger group (age < 49) showed associations between worse sleep quality and more subsequent alcohol use whereas the older group (age > 50) drank more following nights of better sleep quality.^82^

The complex findings in EMA studies speak to the relevance of considering the multidimensional nature of sleep. In one study of undergraduate students who consumed alcohol (mean age = 20.5 years), shorter sleep and earlier wake times (based on actigraphy) and better sleep quality (based on self-report) all predicted more alcohol use the next day.^41^ In the combined model that included all the sleep predictors simultaneously, only waking earlier and better perceived sleep quality upon waking predicted more alcohol use. One interpretation of this is that shortened sleep led to deeper, more consolidated sleep, perceived in turn as higher quality, although it remains possible that shorter sleep may have impacted other intervening mechanisms (e.g., impaired cognitive control). Alternatively, as the authors suggested, late adolescents and young adults may be more likely to socialize and drink when feeling refreshed, especially given that drinking among adolescents and young adults primarily occurs in social contexts.^83^ Collectively, these findings suggest the value of considering multidimensional sleep relationships with alcohol using designs that allow consideration of both short-term (i.e., day-to-day) and longer-term (i.e., months-to-years) timescales, such as embedding an EMA burst design within a longitudinal study, as done by Graupensperger and colleagues.^51^ Relatedly, such designs allow parsing of between-person and within-person effects, which may well reveal distinct sleep–alcohol associations at the between-person and within-person levels.

In summary, the published longitudinal data indicate that multiple sleep and/or circadian characteristics prospectively predict alcohol-related outcomes during adolescence through young adulthood. However, the current literature is limited by overreliance on a relatively small number of longitudinal studies, largely relying on self-report measures, and insufficient consideration of the multidimensional nature of sleep. Important next steps include, but are not limited to, consideration of different timescales, including within the same study design, and examination of key mediators and moderators of sleep–alcohol associations.

### Experimental Sleep and Alcohol Studies

At present, experimental evidence of causal effects of sleep on alcohol-related outcomes is based solely on insomnia treatment studies in individuals with heavy alcohol use and/or AUD, most of which are from samples older than adolescents or young adults. A systematic review and meta-analysis of nine studies of primarily middle-aged adults^7^ concluded that insomnia treatment, particularly behavioral treatment, improved sleep quality and reduced depression in individuals with AUD. The authors found no definitive benefit of insomnia treatment for reducing alcohol use, although the relapse rates in two trials of cognitive-behavioral therapy for insomnia (CBT-I) were considerably lower (11% and 15%) than might be expected for adults in AUD treatment.^84^ Caution is warranted in drawing strong conclusions about the potential impact on alcohol-related outcomes based on these studies, however, as the review also noted limitations related to small samples, relatively short follow-up periods, and not focusing on participants who were concurrently engaged in AUD treatment. These limitations reflect the fact that the studies generally were designed to focus on sleep outcomes rather than alcohol outcomes. Moreover, these studies varied in whether the patients were seeking or engaged in AUD treatment, and whether they were required to be abstinent at study start.

The limited published data from two sleep treatment studies in late adolescents and emerging adults are broadly consistent with the prior literature in adults, suggesting that sleep disturbance in the context of heavy alcohol use is amenable to nonpharmacological interventions; however, it remains unclear whether improving sleep measurably reduces alcohol involvement. A novel web-based intervention including both sleep and alcohol content improved sleep quality and sleep-related impairment in heavy-drinking college students,^85^ although it did not outperform a control condition (psychoeducation about sleep hygiene) and did not significantly improve actigraphy-based sleep outcomes. Interestingly, although alcohol use through a 3-month follow-up declined in both conditions, reductions were larger in the control condition. The results suggested that greater reductions in sleep-related impairment may predict greater reductions in drinking (medium-to-large effect size), but those findings were not statistically significant.

Related work by Fucito and colleagues suggested that heavy-drinking college students were more receptive to sleep-focused interventions (even if they included content related to drinking) than to purely alcohol-focused interventions.^86^ This may be due to less stigma associated with sleep treatment. Aside from direct effects of sleep treatment on alcohol outcomes, this could mean that sleep treatment may provide a “foot in the door” for individuals with sleep and alcohol problems. Accordingly, Fucito and colleagues are currently conducting a sleep intervention trial that focuses on sleep hygiene in young adults ages 18 to 25 who drink heavily.^87^

A more recent study tested the efficacy of CBT-I in 56 young adults ages 18 to 30 who reported monthly binge drinking and met criteria for insomnia disorder.^88^,^89^ The study differed from prior CBT-I and alcohol studies in the sample age and that participants were still actively drinking at the start of CBT-I. The only alcohol-related treatment component was the standard sleep hygiene recommendation to reduce alcohol use before bedtime. With regard to sleep outcomes, CBT-I reduced self-reported insomnia severity relatively better than the sleep hygiene control condition, although neither treatment significantly improved actigraphy-based sleep efficiency.^88^,^89^ Although drinking quantity and drinking-related consequences both decreased over time, these outcomes were not differentially better during CBT-I.^88^ However, although insomnia improvements were not related to changes in drinking, they did mediate the reduction in alcohol-related consequences in the CBT-I group. A secondary analysis reported greater (albeit modest) reductions in alcohol craving for the CBT-I group than for the control group that, again, were statistically mediated by improvements in insomnia.^89^ However, those reductions in alcohol craving were not sustained at the 1-month follow-up assessment.

In summary, the existing experimental literature on sleep predictors of alcohol outcomes during adolescence and young adulthood is confined to a handful of trials testing nonpharmacological sleep interventions in individuals reporting heavy drinking and/or AUD. Consistent with the parallel literature in adult samples, such interventions appear beneficial for sleep-related outcomes but with no clear impact on alcohol-related outcomes. However, a preliminary finding of CBT-I reducing alcohol craving is worth further investigation, as is the further development of sleep-focused treatments, perhaps including more consideration of circadian factors.

### Potential Mediators and Moderators

Prior reviews have examined plausible mechanisms linking sleep/circadian disturbances to alcohol use and alcohol-related problems, with a particular emphasis on reward function.^90^–^92^ A recent review by the authors^91^ proposed a broader conceptual model that considered both positive and negative reinforcement pathways, and noted that elevated impulsivity may exacerbate either pathway. While this model may have heuristic value, it is not without limitations. These include not explicitly addressing bidirectional effects (i.e., alcohol effects on sleep/circadian function) or incorporating plausible factors that influence which pathway is most salient for a particular individual or at a given time. Further, research on sleep–alcohol associations has largely been conducted with samples of predominantly White-identifying individuals and has largely not explored possible differences in associations between sleep and alcohol across assigned sex, racial and ethnic identities, and for LGBTQIA+ individuals. The following sections offer some preliminary evidence of the importance of including diverse samples in future investigations and of examining differences in associations to ensure generalizability of future treatments and to inform culturally responsive interventions for both sleep/circadian disturbances and AUD.

#### Mechanisms related to positive reinforcement

Extensive cross-sectional, longitudinal, and experimental evidence from both human and preclinical studies has supported the influence of sleep/circadian factors on reward-related processes and underlying physiology^92^ and, in turn, the relevance of reward-related processes to risk for alcohol use and related problems.^28^,^93^,^94^

Although relevant human experimental studies probing sleep/circadian effects on reward-related processes have been more scarce than animal models, experimental sleep deprivation protocols have demonstrated causal effects on reward-related brain function in healthy adolescent and young adult samples.^95^–^97^ For example, experimentally imposed circadian misalignment reduced the neural response to monetary reward and during response inhibition in healthy adolescents without regular substance use.^98^ The analyses included objective measures of sleep duration and alertness, thus suggesting circadian effects on reward function beyond those of insufficient sleep. However, these studies have focused on non-alcohol rewards. In contrast to emerging animal research suggesting circadian misalignment during adolescence alters reward circuitry function and increases alcohol use during adulthood,^50^ almost no published human studies have examined sleep/circadian effects on alcohol cue reactivity and/or its neural correlates. Furthermore, few existing studies have combined sleep/circadian effects, reward, and alcohol outcomes, although one cross-sectional analysis found that “eveningness”—the self-reported preference for relatively later timing of sleep and activity—was associated with altered neural processing of reward, which in turn is associated with greater alcohol use and AUD symptoms.^99^ A longitudinal analysis in the same study found that the prospective association between eveningness and AUD symptoms was statistically mediated by the medial prefrontal cortex response to monetary reward.^52^ Most recently, a study reported that an objective measure of circadian misalignment (measured on a Thursday) prospectively predicted a lower neural response to monetary reward (measured on a Friday) in late adolescents with regular alcohol use.^100^ However, the reduced neural response to reward did not prospectively predict alcohol use that weekend, but rather was associated with more binge drinking episodes at baseline. Finally, in the aforementioned CBT-I trial in adolescents and young adults reporting heavy alcohol use and insomnia, the investigators found evidence of relatively larger reductions in delay discounting (large rewards only) in the CBT-I group, although this was not mediated by insomnia severity. However, there was no apparent effect on negative affect, suggesting that improved sleep may have relatively greater effects on reward-related processes.^89^

Some evidence suggests sleep/circadian modulation of the stimulating effects of alcohol (e.g., increases in energy and excitement). This may be particularly relevant during adolescence, when alcohol may be relatively more stimulating and less sedating than in adulthood.^101^–^103^ Notably, a relatively more stimulating response to alcohol is a risk factor for AUD. Thus, adolescents at high risk for AUD endorsed greater alcohol-induced stimulation and stronger wanting for alcohol compared to adolescents at low risk for AUD.^104^ Moreover, young adults reporting greater stimulation after alcohol administration were more likely to have developed AUD by 10-year follow-up.^105^ In laboratory-based sleep studies in late adolescents and emerging adults, acute alcohol administration did not reduce SOL,^36^ especially when consumed in the evening,^106^ suggesting the stimulating rather than sedating effects also may be influenced by time of administration. Furthermore, later sleep timing was associated with greater self-reported stimulation response following alcohol administration in the laboratory (at least in White male participants).^107^

Lastly, sleep/circadian factors may be relevant to positive reinforcement-related alcohol cognitions. Adolescents and young adults tend to report more motives attributed to improving their social experiences and enhancing enjoyment versus motives related to attenuating negative affect (i.e., coping).^108^ Given that eveningness is associated with increased alcohol motives across the board,^109^ including enhancement and social motives, it is possible that the tendency toward later sleep/circadian timing in this age group contributes to reasons for using alcohol.

#### Mechanisms related to negative reinforcement

Adverse life events and stress levels disrupt sleep and prospectively predict AUD outcomes, both on a longitudinal basis during adolescence into adulthood,^110^,^111^ and more proximally (day to day).^112^,^113^ Furthermore, demonstrating sleep- or drinking-related reactivity to stress heralds the risk for sleep-114 or alcohol-related problems^115^ in the future.

Several lines of evidence indicate that sleep problems, perhaps driven by stress and/or anxiety, may lead to using alcohol as a coping method, thus implicating negative reinforcement pathways. Studies suggest that about 10% (range 6% to 16%) of adolescents and young adults report using alcohol as a sleep aid, with higher rates in individuals with heavier alcohol use and/or worse sleep.^116^–^118^ Interestingly, one longitudinal study of adolescents with and without AUD found that their use of alcohol as a sleep aid declined over time, dropping by half from baseline to 5-year follow-up; this may reflect adolescents’ learning that alcohol’s effectiveness at promoting sleep declines with regular use.^119^

Compared to “good sleepers,” adults with insomnia may experience relatively greater tension reduction and deeper sleep (based on slow-wave sleep) in response to alcohol, underscoring why they might initially turn to alcohol as a sleep aid. Although experimental evidence suggests they rapidly develop tolerance to these effects, these individuals often persist in choosing alcohol as a sleep aid.^120^,^121^ Similarly, young adults with insomnia who regularly use alcohol reported better sleep efficiency on drinking days, seemingly due to shorter SOL, in a recent EMA study,^42^ and reported sleeping worse on nights when they avoided alcohol in the 2 hours before bed.^122^ In contrast with the experimental study,^120^ the association with better sleep efficiency remained even after accounting for number of consecutive drinking days.^42^ Notably, these associations were not observed for actigraphy-based sleep efficiency.

Sleep also may modulate effects of stress on alcohol use. Along with associations with drinking motives in general (see above), eveningness in college students was associated with worse coping with stress, which in turn may predict drinking to cope.^109^ Another study found that late chronotypes had both more adverse childhood experiences and greater alcohol use during young adulthood.^123^

#### Craving-related mechanisms

Craving—a criterion for diagnosis of AUD and widely studied as a proximal predictor of alcohol use—is a complex construct, with apparent contributions of both positive- and negative-reinforcement processes.^124^ Recent studies have offered preliminary evidence that alcohol craving is influenced by sleep/circadian factors. Two studies reported the presence of a 24-hour rhythm in alcohol craving,^125^,^126^ suggesting modulation by circadian rhythms, although the studies were mixed in whether sleep characteristics predicted the timing or amplitude of the craving rhythm. Lower sleep quality was associated with elevated tonic (i.e., long-term) craving as determined using the Obsessive-Compulsive Drinking Scale, but not with cue-induced craving (as measured using the Alcohol Urge Questionnaire) during a cue reactivity paradigm in patients with AUD.^127^ Finally, less sleep predicted more alcohol craving the next day in an EMA study,^51^ and reductions in insomnia severity mediated reductions in alcohol craving in a CBT-I trial.^89^

Relatedly, growing evidence implicates a role for the orexin/hypocretin system in sleep-alcohol associations via both negative reinforcement and reward-related processes. Orexin/hypocretin regulates wakefulness, reward seeking, and other motivated behavior, including alcohol craving and alcohol seeking; in turn, the orexin/hypocretin system is modulated by acute and chronic stress.^128^,^129^ Ongoing trials are testing whether suvorexant, a dual orexin receptor antagonist, can reduce both alcohol craving and insomnia symptoms.^130^,^131^

#### Impulsivity-related mechanisms

Similar to craving, the multifaceted construct of “impulsivity” may be relevant to both positive and negative reinforcement pathways in understanding sleep/circadian-related risk for alcohol involvement. In general, facets of impulsivity are considered a key risk factor for the development of heavy alcohol use and related problems.^29^,^32^ Importantly, impulsivity facets may differentially relate to alcohol use through both positive and negative reinforcement pathways. For example, negative urgency, or acting rashly in response to strong negative mood, may reflect drinking to cope with negative mood/stress whereas positive urgency may reflect expecting alcohol to increase arousal.^132^

Multiple sleep/circadian characteristics have been linked to impulsivity domains (e.g., Kang et al.^34^,^35^). For example, recent prospective evidence in adolescents suggested that both sleep duration and insomnia were bidirectionally associated with impulse control.^133^ Recent studies found that later chronotype was associated with greater impulsivity overall (e.g., Kang et al.^34^), including greater self-reported trait- and state-level impulsivity across multiple subdimensions in White male drinkers.^134^ Also, as noted above, experimentally imposed circadian misalignment reduced neural activation in the right inferior frontal gyrus during response inhibition in healthy and non–substance-using adolescents.^98^

#### Moderation by assigned sex and gender identity

Studies found that both sleep/circadian characteristics and risk for problematic alcohol use vary by assigned sex at birth (sex); however, there has been insufficient attention to the role of sex in sleep/circadian–alcohol associations. This is important as rates of AUD among female individuals have risen 84% in the past decade, compared to a 34% increase among male individuals.^135^,^136^ Consistent with this trend, alcohol use has risen for women but not men.^137^ Prior research found that female individuals reported higher levels of disturbed sleep (e.g., insomnia),^138^ while male individuals tended to report later sleeping times.^139^ Recent findings suggest that sleep/circadian characteristics differentially contribute to alcohol risk for male and female individuals. Indeed, recent longitudinal studies found that male individuals in particular may be at heightened alcohol-related risk attributed to sleeplessness^138^,^140^ and later weekday/weekend bedtime.^6^ However, other studies observed stronger associations between multiple sleep characteristics (e.g., total sleep time, sleep efficiency, nighttime awakenings) and alcohol-related risks among female individuals.^5^,^141^ Factors that may contribute to increases in alcohol use and sleep disturbance among female individuals may include heightened drinking to cope with negative affect and stress.^142^–^144^ However, these studies did not clarify whether they were measuring assigned sex or gender identity (the term “identity” is used to reflect that race and gender are social constructs^145^ and that the vast majority of research on humans asks participants to self-identify their race and gender).

Inequities in sleep^146^,^147^ and alcohol use^148^ exist for individuals with minoritized gender identities (e.g., transgender, nonbinary, gender-fluid). Importantly, a recent study examining factors that influenced sleep among individuals who identified as transgender found that one-third of the sample endorsed feelings of internalized shame (i.e., distress, anxiety, and dysphoria attributed to their identity) as reason for sleep disturbance.^149^

Inequities in sleep duration^150^ and alcohol use^151^ also exist among individuals with minoritized sexual orientations (e.g., lesbian, gay, queer, bisexual). However, only one cross-sectional study has examined whether sleep/circadian characteristics contribute to inequities in alcohol problems and whether these associations present differently among subgroups of people with minoritized sexual orientations (e.g., bisexual women, gay men).^152^ The study found that compared to heterosexual men, gay men were less likely to experience short sleep duration and reported consuming fewer alcohol drinks per day. Lesbian and bisexual women, when compared to heterosexual women, reported a greater number of alcoholic drinks per day and were more likely to use sleep medication. Further, bisexual women were more likely to experience short sleep duration and to be diagnosed with a sleep disorder compared to heterosexual women.

It is important to place these findings within a minority stress model framework, where individuals with minoritized identities are exposed to identity-based stressors^153^ that occur at both interpersonal and systemic levels.^154^ Identity-based stressors—defined as chronic modes of stress attributed to discrimination and internalized stigma directed at one’s minoritized identity (e.g., sexual, gender, or racial identities)—are prominent predictors of health inequities, including alcohol behaviors and sleep disturbances, among individuals with minoritized sexual and gender identities.^146^,^155^,^156^ However, further examination of possible differential associations between sleep indices and alcohol behaviors is needed.

#### Moderation by racial and ethnic identities

As a function of sociohistorical context and multiple levels of discrimination, inequities in sleep health and alcohol problems have been shown for individuals with minoritized racial and ethnic identities.^157^–^162^ Significantly less research has examined if sleep disturbances related to discrimination contribute to the inequities in alcohol problems and whether the associations between sleep and alcohol differ among individuals with different racial or ethnic identities. Structural racism affects neighborhood-level factors that impact sleep (e.g., noise pollution) and alcohol use (e.g., alcohol outlet density), and neighborhood socioeconomic indicators (i.e., income, crime rates, discrimination) have been implicated in inequities in sleep, which may contribute to downstream poor health outcomes.^163^ Specifically, studies have identified that individuals with low socioeconomic status tend to inhabit urban areas, which may be more hazardous and noisier and may have higher levels of crime. Such neighborhood characteristics have been found to be associated with greater rates of chronic sleep disturbance,^164^ which in turn have been linked to heightened alcohol consumption among adolescents as reviewed above (also see Edwards, Reeves, and Fishbein^165^). As individuals with minoritized racial and ethnic identities may be more socioeconomically disadvantaged as a result of sociohistorical structural and interpersonal discrimination, these youth may be at greater risk for poor sleep quality in addition to elevated risk for alcohol use. These environmental factors may also affect associations between sleep and alcohol differently for individuals with minoritized racial or ethnic identities. All of these potential associations have direct implications for prevention and treatment.

Cross-sectional evidence suggests that alcohol use may be more disruptive to sleep for Black individuals relative to White individuals. Among men with AUD, Black men had more severe sleep disturbances compared to White men.^166^ Based on National Health Interview survey data collected between 2004 and 2015, sleep duration and sleep quality were highest in Black individuals who never consumed alcohol (i.e., lifetime abstention) and worsened as alcohol use involvement increased.^167^ For White individuals, this pattern was more variable. Importantly, the racial differences in this study were more pronounced for women than men, demonstrating the importance of examining intersectionality.

Research examining associations between sleep and alcohol use in minoritized racial or ethnic groups beyond Black or African American individuals is nascent. However, consistent with research with predominantly White samples, binge drinking in adolescence has been shown to relate to poorer sleep quality in young adulthood for Mexican American and American Indian (as defined in the article) individuals.^168^

Studies examining how sleep may differentially affect alcohol use and experiences while drinking across racial and ethnic groups are even more sparse. Preliminary research found that later sleep timing was related to increased sensitivity to the stimulating effects of alcohol for White men but not Black men;^107^ however, no differences existed in associations with 24-hour rhythms in alcohol craving for Black and White young adults.^125^

#### Other possible moderators

Multiple other moderators of the relationship between sleep/circadian factors and alcohol use are plausible but have received little attention to date, including the role of age and/or developmental stage. An exploratory analysis of the longitudinal data from the National Consortium on Alcohol and Neurodevelopment in Adolescence study^5^ found a different pattern of sleep/circadian predictors of binge alcohol severity at middle- and high-school age time points versus post–high-school age time points. This difference could reflect context, given systematic early school start times versus more flexibility in schedules after high school (i.e., college and/or employment), but more research is needed to replicate and further clarify this finding.

Sleep/circadian-related risk for alcohol outcomes also may be moderated by the stage of alcohol use and related problems, potentially varying as individuals progress through the three-stage cycle framework of AUD—binge/intoxication, negative affect/withdrawal, and preoccupation/anticipation as described by Koob and Colrain.^2^ The shift from enhancement motives/positive reinforcement in the binge/intoxication phase to coping motives/negative reinforcement in the withdrawal/negative affect stage could be paralleled by a shift in relevant sleep/circadian pathways. That is, accumulating alcohol use/problems may contribute to more chronic and/or more distinct sleep/circadian disturbances, which in turn may maintain or exacerbate alcohol involvement. Additionally, sleep problems have been identified as a risk factor for relapse during early abstinence in individuals with AUD.^2^

## Conclusions and Future Directions

Based on the above discussion, future research on the intersection between sleep and alcohol should address existing gaps related to both research methodology and specific questions addressed. For example, future studies should employ assessment batteries able to assess multidimensional sleep/circadian characteristics and should include both self-report and objective measures, particularly objective assessments not yet sufficiently leveraged in this literature, such as the Multiple Sleep Latency Test to assess daytime sleepiness. Research also can benefit from the use of combined longitudinal and intensive longitudinal designs, such as EMA bursts within a larger longitudinal study framework, which will allow consideration of both different timescales and parsing of between-person (trait) and within-person (state) effects.

Such studies should further explore the role of relevant moderators, with particular attention to sleep–alcohol associations for individuals with minoritized identities. Equally important is consideration of the association between sleep and cannabis use, including simultaneous use with alcohol, given the high prevalence of this practice in late adolescents and young adults and evidence suggesting somewhat opposing effects of both substances on sleep. Examination of potential differences in sleep–alcohol associations across international samples could help determine how varying cultural contexts may differentially influence sleep, alcohol use, and their association.

Furthermore, experimental research is needed to demonstrate causal effects of sleep/circadian manipulations on alcohol-related risk. Additionally, experimental studies using approaches such as forced desynchrony or ultradian sleep-wake protocols could help parse the role of circadian versus sleep homeostatic contributions in modulating alcohol-related processes (e.g., alcohol craving).

Other research gaps to be addressed include the clarification of potential shared genetic variance and/or pleiotropic contributions to sleep–alcohol associations, which should further clarify trait- versus state-level effects, as well as investigation of different mechanistic pathways linking sleep to alcohol outcomes. These ideally should allow for comparison of distinct pathways within the same dataset and include not only the putative mechanisms described above (e.g., reward function, negative reinforcement, impulsivity) but also others that may well be worth consideration, such as hypothalamic-pituitary-adrenal axis function.

Finally, research gaps exist with respect to treatment of adolescents and young adults with both alcohol problems and sleep problems. Rigorous treatment studies in this population are needed that go beyond CBT-I to include attention to circadian factors, and with sufficient follow-up periods to better elucidate differential effects on alcohol.

Overall, the existing longitudinal and experimental evidence indicates that a range of sleep/circadian characteristics during adolescence and young adulthood influence risk for the development of alcohol use and/or related problems. Although studies in late adolescents and young adults engaging in regular and/or heavy drinking show that sleep treatment can improve sleep in those individuals, as well as potentially reduce alcohol craving and alcohol-related consequences, no studies in any age group have yet demonstrated that improving sleep reduces drinking behavior. Future research embedding intensive longitudinal studies within prospective research studies is needed to understand the underlying mechanistic pathways from sleep and circadian rhythm to differential alcohol use behaviors and problems as there is evidence that specific sleep indices may relate to certain AUD criteria.^169^ Such studies could hold promise for informing treatment for both sleep problems and AUD.

## Figures and Tables

**Table 1:** Summary of Longitudinal Studies Including Sleep-Related Predictors and Alcohol-Related Outcomes in Adolescent and/or Young Adult Samples

		Sample		Study Design		Multidimensional Sleep Variables—Related to Alcohol Outcome(s)?								
Author	Year	Sample Size & General Description	Sample Demographics*	Design	Time Frame	Regularity	Satisfaction†	Alertness	Timing	Efficiency	Duration	Composite	Alcohol Finding	Tested Sex/Gender and/or Race/Ethnicity
Fucito et al.^41^	2018	42 college students with concerns about their sleep, ≥ 1 occasion of heavy drinking in the past month	*M* age = 20.5 (*SD* = 1.31); 48% female; 4% Asian, 5% Black, 5% more than one race, 2% other, 69% White; 19% Hispanic/Latino	Intensive longitudinal; 7day protocol	Days	-	Y	Y	Y	-	-	-	Shorter sleep duration (actigraphy), earlier wake time (actigraphy), and better sleep quality all predicted more drinks the following day. In full model including all sleep variables, only wake time, sleep quality, and alertness upon waking predicted later alcohol use.	Tested differences across gender identity in sleep and alcohol variables (none significant)
Graupensperger et al.^51^	2022	409 young adults reporting past-month simultaneous use of alcohol and cannabis use and drinking alcohol ≥ 3 times in past month	*M* age = 21.6 (range = 18–25); 51% female; 16% Asian, 4% Black, 11% more than one race, 4% other, 48% White; 16% Hispanic/Latino	Intensive longitudinal; five 14-day bursts of twice-daily surveys separated by 4 months (i.e., 70 days total across 16 months)	Days, months, years	-	-	-	-	-	Y	-	At burst-level, shorter sleep duration was associated with greater alcohol use. At all three levels (person, burst, and daily), shorter sleep duration was associated with stronger morning alcohol craving. At burst and daily levels, shorter sleep duration was associated with stronger afternoon alcohol craving.	None tested
Hasler et al.^170^	2014	696 participants from study at the Pittsburgh Adolescent Alcohol Research Center. At baseline, 347 participants with current AUD (AUD+); 349 participants with no current or past AUD (AUD−)	AUD+: *M* age = 16.7 (range = 12–19); 37% female; < 1% Asian, 12% Black, 87% White AUD−: *M* age = 15.8 (range = 12–19); 57% female; < 1% Asian, 25% Black, < 1% Native American, 75% White	Longitudinal; data from baseline, 1-, 3-, and 5-year follow-up assessments	Years	Y	Y	-	-	-	N	-	In AUD− group, greater insomnia at baseline predicted increase in AUD symptoms at 1-year follow-up, while greater variability in weekday-weekend sleep duration predicted increases in AUD symptoms at 3- and 5-year follow-up.	AUD group differences reported across racial groups
Hasler et al.^54^	2016	707 children in Center for Education and Drug Abuse Research (CEDAR) study	*M* age = 11.4 (range = 9–13); 28.9% female; 21% Black, 79% White	Longitudinal; data from eight assessments spaced 2–3 years apart. Last assessment at approximately age 30	Years	Y	Y	-	-	-	-	-	More restless sleep predicted earlier onset of alcohol use. More variable sleep timing predicted earlier onset of AUD.	Sleep differences reported across racial groups; no significant differences across sex
Hasler et al.^53^	2017	729 adolescents in National Consortium on Alcohol and Neurodevelopment in Adolescence (NCANDA) study	*M* age = 15.9 (range = 12–21); 51% female; 7% Asian, 12% Black, 41% other, 75% White; 12% Hispanic/Latino	Accelerated longitudinal design; data from baseline and 1–year follow-up assessments	Months	N	N	N	Y	-	Y	-	Greater eveningness, later bedtime (weekday and weekend), and shorter weekday sleep duration all predicted higher severity of binge alcohol use.	Sleep differences reported across racial and ethnic groups
Hasler et al. 52	2017	93 male participants from Pitt Mother & Child Project originally recruited in infancy	Assessments at age 20 and age 22; race/ethnicity not reported	Longitudinal; data from two time points (age 20 and age 22)	Years	-	-	-	Y	-	-	-	An indirect path was observed from circadian preference at age 20 to alcohol use and dependence at age 22 via the neural (medial prefrontal cortex) response on a monetary reward task. However, circadian preference at age 20 did not directly predict alcohol use or dependence at age 22.	None tested
Hasler et al.^5^	2022	831 adolescents in NCANDA study	*M* age = 16.2 (range = 12–21); 50.9% female; 7% Asian American, 12% Black, 41% other, 75% White; 12% Hispanic/Latino	Accelerated longitudinal design; data from baseline through 5-year follow-up (six annual assessments)	Years	N	N	Y	Y	-	Y	-	Greater eveningness, more daytime sleepiness, later weekend sleep timing, and shorter sleep duration (weekday/weekend) all predicted more severe alcohol binge drinking the following year. Sleep associations with binge severity differed between middle/high school versus post-high school adolescents.	Sleep differences reported based on sex; no differences across racial groups were assessed
Haynie et al.^67^	2018	2,785 high school students from the NEXT Generation Health Study	Age not reported; 55% female; 14% Black, 5% Other, 62% White; 19% Hispanic/Latino	Longitudinal; data from 10th, 11th, and 12th grades (Waves 1–3)	Years	Y	-	-	Y	-	N	-	Later chronotype and greater social jet lag both predicted greater likelihood of alcohol use and heavy episodic drinking (Wave 2 to Wave 3 only). Sleep duration did not predict subsequent alcohol involvement.	Sleep differences reported across gender identity and racial and ethnic groups in Supplement
Ho et al.^69^	2020	1,678 adolescents from Taiwan Youth Project; participants who had ever smoked cigarettes or consumed alcohol before Year 1 were excluded	*M* age = 13; 50% female; 8% Hakka, 12% Mainlanders, 1% Original Residents, 2% Other, 88% Weinan Islanders	Longitudinal; cohort starting in 7th grade (Year 1, 2000) and assessed until age 21 (Year 9, 2009); five waves of assessment	Years	Y	N	-	-	N	Y	-	Accumulating waves of sleep duration (< 6 hours) and social jet lag (≥ 0.5, 1, or 2 hours) increased the frequency of alcohol use at age 21. Sleep disturbance and sleep duration of at least 7 or 8 hours were not predictive of alcohol use.	None tested
McGlinchey & Harvey^55^	2015	4,882 adolescents from ADD Health	Wave 2: *M* age = 16; Wave 3: *M* age = 21.8; 52% female; 5% Asian, 24% Black, 4% Native American, 59% White; 8% Hispanic/Latino	Longitudinal; data from Wave 2 (1996) and Wave 3 (2001–2002)	Years	-	-	-	Y	-	-	-	Later bedtime at Wave 2 predicted increased odds of reporting alcohol “abuse” at Wave 3.	Alcohol use differences reported across sex and racial groups, although specific differences across racial groups were not specified
Mike et al.^56^	2016	186 boys from Pitt Mother and Child Project	*M* age = 11 (at baseline); follow-up between ages 20 to 22; 45% “non-White,” 55% White	Longitudinal; data from baseline (age 11), with alcohol use history assessed at age 20 and age 22	Years	-	Y	-	-	-	Y	-	Shorter sleep duration and lower sleep quality both predicted earlier alcohol use, intoxication, and repeated use.	None reported
Miller et al.^171^	2016	568 college students who had violated campus alcohol policy and had been mandated to an alcohol prevention intervention	*M* age = 19.2 (*SD* = 1.2); 28% female; 84% White	Longitudinal; data from baseline, 1-, 3-, and 5-month follow-up assessments	Months	-	-	-	-	-	Y	-	Lower baseline sleep adequacy predicted alcohol-related consequences at 1-month follow-up. A stronger association was found between drinks per week and alcohol-related consequences in individuals with lower baseline sleep adequacy.	None tested
Miller et al.^57^	2017	829 middle school students from Rhode Island study examining risk factors for initiation/progression of drinking	*M* age = 12.6 (*SD* = 1.02); 52% female; 15% “non-White;” 12% Hispanic/Latino	Longitudinal; ongoing prospective Web-based survey over 4-year period, with recruitment beginning in 2009; data from 6-month assessments over 2 years (Times 2–5), 3-year follow-up (Time 6), and 4-year follow-up (Time 7); two sleep assessments within first 2 years of study	Years	N	-	Y	-	-	Y	-	Both shorter sleep and greater daytime sleepiness predicted onset of full drinking, heavy episodic drinking, and alcohol-related consequences. Weekend bedtime delay predicted alcohol outcomes.	Racial identity dropped from model due to nonsignificant relation to alcohol outcomes.
Miller et al.^42^	2021	56 young adults; reporting ≥ 1 binge episode in past 30 days; also meeting diagnostic criteria for insomnia	*M* age = 22.4 (*SD* = 2.7); 75% female; 5% Black, 11% multiracial, 2% Native American/Native Alaskan, 82% White; 4% Hispanic/Latino	Intensive longitudinal design; online survey and actigraphy for at least 7 days (average 8.52 diaries; range = 1–15 days)	Days	-	Y	-	N	Y	N	-	Better self-reported sleep efficiency predicted greater drinking the next day.	Differences based on sex were not reported; differences across racial/ethnic groups were not tested.
Nguyen-Louie et al.^58^	2018	95 adolescents from neuroimaging study on adolescent substance use in San Diego	Time 1: *M* age = 13.4 (*SD* = 0.7); Time 2: *M* age = 15.1 (*SD* = 0.9); Time 3: *M* age = 19.8 (*SD* = 0.9); 47% female; 6% Asian, 3% Black, 17% multiracial, 5.3% other, 68% White; 20% Hispanic/Latino	Longitudinal; data from Time 1 (ages 12–14), Time 2 (~ 1.5 years after Time 1), and Time 3 (~ 2.6 years after Time 2)	Years	-	-	Y	Y	-	-	Y	More frequent erratic sleep/wake behaviors (composite variable of delayed/variable bedtime and daytime dysfunction), greater sleepiness, and greater eveningness at Time 2 generally predicted more lifetime alcohol use by Time 3. However, erratic sleep/wake behaviors predicted lower alcohol use after controlling for daytime sleepiness and chronotype. Sleep variables were also tested as mediators between Time 1 risk factors (family history of SUD, inhibitory control, and internalizing/externalizing) and Time 3 lifetime alcohol use. Only erratic sleep/wake behaviors mediated Time 1 inhibitory control on Time 3 alcohol use.	None tested
Pasch et al.^59^	2012	723 adolescents from Identifying the Determinants of Eating and Activity (IDEA) or Etiology of Childhood Obesity (ECHO) cohort studies	*M* age = 14.7 (range = 10–17); 51% female; 1% Asian, < 1% American Indian, 5% Black, < 1% Native American/Pacific Islander, 6% other, 86% White; 5% Hispanic/Latino	Longitudinal; both 2-year studies; baseline data collected in 2006–2007 (IDEA) and 2007–2008 (ECHO); follow-up data collected in 2008–2009 (IDEA) and 2009–2010 (ECHO)	Years	Y	-	-	-	-	N	-	Larger weekend bedtime delay at baseline predicted higher likelihood of alcohol use at follow-up.	Sleep differences were reported across sex and racial groups.
Pieters et al.^60^	2015	555 adolescents from the Netherlands	*M* age = 13.96 (range = 11–16); 52% female; 86% had two White parents, 10% had a mix of White and “non-White” parents, 4% had two White parents	Longitudinal; two waves: Wave 1 (early 2008) and Wave 2 (late 2008)	Months	-	-	-	-	-	-	Y	Sleep problems at Wave 1 positively predicted alcohol use at Wave 2.	None tested
Stephenson et al.^72^	2020	3,402 participants (1,435 complete twin pairs; 36% monozygotic) from FinnTwin12, a population-based study of Finnish twins born between 1937 and 1987	Time 1: *M* age = 12; Time 2: *M* age = 14; Time 3: *M* age = 22 (range = 20–26); 57% female; no information on race/ethnicity	Longitudinal; analyses focus on predictors from waves at ages 12 and 14 predicting young adult outcomes (age 22)	Years	-	-	-	-	Y	-	-	At individual level, sleeping problems at ages 12 and 14 predicted alcohol misuse in young adulthood, but were no longer significant in co-twin comparisons.	None tested
Tavernier et al.^78^	2014	780 first-year Canadian university students identifying as being morning- or evening-type	*M* age = 19 (*SD* = .09); 72% female; 87% domestic-Canadian, remaining international students (37% Asia, 10% Caribbean, 15% European Union); race not reported	Longitudinal; 2-year study: Time 1 (baseline) and Time 2 (1-year follow-up)	Years	-	-	-	-	-	-	Y	Composite variable based on chronotype, insomnia symptoms, and sleep duration. Evening-type subgroups generally reported more alcohol use than morning-type subgroups, although evening-poor sleep and morning-poor sleep subgroups did not differ. The evening subgroups (good/moderate/poor sleep) did not differ in alcohol use.	Did not test differences across racial groups; similar distribution of morning-/evening-types based on sex
Troxel et al.^6^	2021	3,265 youth from southern California study by RAND Corporation	Wave 6: *M* age = 16.2 (*SD* = 0.7); Wave 11: *M* age = 21.6 (*SD* = 0.8); 53% female; 20% Asian; 2% Black (non-Hispanic); 12% other/multiracial; 20% White (non-Hispanic); 47% Hispanic/Latino	Longitudinal; data from six waves: Wave 6 (May 2013 to April 2014) to Wave 11 (July 2018 to June 2019)	Years	Y	Y	-	Y	-	N	-	Greater trouble sleeping, later weekday and weekend bedtimes, and smaller reductions in social jet lag were associated with higher likelihood of alcohol use over time.	Differences were reported in the associations between weekday/weekend bedtime and alcohol use based on sex.
Troxel et al.^68^	2022	2,995 youth from southern California study by RAND Corporation	Wave 8: *M* age = 18.3 (*SD* = 0.8); Wave 13: *M* age = 23.6 (*SD* = 0.8); 54% female; 20% Asian, 2% Black (non-Hispanic), 12% other/multiracial, 20% White (non-Hispanic); 46% Hispanic/Latino	Longitudinal; data from six waves: Wave 8 (June 2015 to May 2016) to Wave 13 (July 2020 to July 2021)	Years	-	-	-	-	-	-	Y	“Good sleepers” (composite variable based on sleep quality, duration, and social jet lag) reported lower levels of alcohol use and consequences. When compared to “suboptimal sleepers,” “good sleepers” also reported less of an increase in alcohol consequences over time.	Differences were reported in composition of classes in terms of sex and ethnicity.
Waddell & Sasser^24^	2022	4,347 participants from ADD Health	Wave 2: *M* age = 16.0 (*SD* = 1.4); Wave 3: *M* age = 21.4 (*SD* = 1.4); Wave 4: *M* age = 28.5 (*SD* = 1.4); 52% female; 4% American Indian/Alaskan Native, 4% Asian, 24% Black, 6% other/multiracial, 68% White; 12% Hispanic/Latino	Longitudinal; data from three waves: Wave 2 (1996), Wave 3 (2001–2002), Wave 4 (2007–2008)	Years	-	-	-	-	-	Y	-	Longer sleep duration was associated with lighter drinking at the between-person level. Increases in sleep duration were associated with decreases in drinking at the within-person level. At the between-person level, higher levels of lack of premeditation were associated with greater drinking. At the within-person level, increases in sensation seeking were most strongly associated with increases in drinking for those reporting decreases in sleep duration.	None tested
Wong et al.^61^	2004	257 boys from Michigan Longitudinal Study (MLS); 60% of participants had parent with lifetime AUD	Wave 1: Age range = 3–5; Wave 2: Age range = 6–8; Wave 3: Age range = 9–11; Wave 4: Age range = 12–14; 100% White	Longitudinal; data from four regular waves at 3–year intervals	Years	-	Y	Y	-	-	-	Y	Composite index of sleep problems predicted higher likelihood of drinking at ages 12–14. Weaker associations were found for individual sleep items (trouble sleeping and overtiredness), although both predicted onset of drinking, but not drunkenness.	Not applicable
Wong et al.^63^	2009	386 children from MLS; 75% of participants with parent who met lifetime AUD	Wave 1: Age range = 3–5;* Wave 2: Age range = 6–8; Wave 3: Age range = 9–11; Wave 4: Age range = 12–14; Wave 5: Age range = 15–17; 24% female; 100% White *Note: Girls joined study between the ages of 6 and 11.	Longitudinal; data from five regular waves and seven annual waves at 3-year intervals	Years	-	-	-	-	-	-	Y	Sleep problems during Waves 1 and 2 increased the probability of onset of alcohol use between the ages of 8 and 14 in boys but did not predict onset of alcohol use in girls before the age of 14. In contrast, sleep problems during Waves 1 and 2 increased the probability of onset of alcohol use between the ages of 15 and 17 in girls, but not boys.	Differences reported in sleep and alcohol outcomes based on sex; differences across racial groups could not be tested
Wong et al.^62^	2010	386 children from MLS; 75% of participants with parent who met lifetime AUD	Wave 1: Age range = 3–5;* Wave 2: Age range = 6–8; Wave 3: Age range = 9–11; Wave 4: Age range = 12–14; Wave 5: Age range = 15–17; 24% female; 100% White *Note: Girls joined study between the ages of 6 and 11.	Longitudinal; data from five regular waves and seven annual waves at 3–year intervals	Years	-	N	Y	-	-	-	-	Overtiredness at ages 3–8 predicted all four alcohol variables (i.e., binge drinking, blackouts, driving under the influence, and alcohol problems) in emerging adulthood (ages 18–20). Sleep during adolescence (age 11–17) did not predict any alcohol outcomes during emerging adulthood.	Sleep differences tested by sex, though none were significant
Wong et al.^172^	2015	6,504 adolescents and young adults from ADD Health	Time 1: *M* age = 16.0 (*SD* = 1.8); Time 2: *M* age = 16.0 (*SD* = 1.6); Time 3: *M* age = 21.8 (*SD* = 1.8); Race/ethnicity/sex not reported	Longitudinal; data from three time points: Time 1 (1994–1995), Time 2 (1996), and Time 3 (2001–2002)	Years	-	Y	-	-	-	Y	-	Sleep duration at Time 1 was negatively associated with binge drinking at Time 2 (a 1-hour increase in sleep was associated with a 9% decrease in the odds of binge drinking). Sleep difficulties at Time 1 were associated with regretted sexual activities due to drinking at Time 2.	Differences reported in alcohol outcomes across sex and racial groups
Zhang et al.^65^	2011	1,611 children from Hong Kong study concerning childhood sleep problems	*M* age (baseline) = 9.0 (*SD* = 1.8); *M* age (follow-up) = 13.7 (*SD* = 1.8); 51% female; racial breakdown not specified	Longitudinal; 5-year study beginning in 2003–2004 (baseline), with follow-up assessment conducted between 2008 and 2010	Years	-	Y	-	-	-	-	-	New incidence of chronic insomnia was associated with increased risk of frequent alcohol use at 5-year follow-up, but baseline chronic insomnia and persistent chronic insomnia were not statistically significant.	Sleep differences reported by sex; differences across racial groups could not be tested.

* Age was rounded to 1 decimal place; demographic (sex/gender and race/ethnicity) percentages were rounded to whole numbers. No studies clarified whether assigned sex at birth or gender identity was assessed and reported. Therefore, the language (e.g., sex vs. gender) in the original article was retained. When possible, race/ethnicity terms were standardized to be consistent with National Institutes of Health categories. In some cases, the published papers did not specify the racial/ethnic identities beyond “White” and “non-White” (or “Caucasian” and “non-Caucasian,” for which “White” and “non-White” were substituted).

† Sleep variables based on insomnia symptoms without numerical data to calculate efficiency were categorized under “Satisfaction.”

*Note:* ADD Health, National Longitudinal Study of Adolescent to Adult Health; AUD, alcohol use disorder; ECHO, Etiology of Childhood Obesity studies; IDEA, Identifying the Determinants of Eating and Activity; *M*, mean; MLS, Michigan Longitudinal Study; NCANDA, National Consortium on Alcohol and Neurodevelopment in Adolescence; *SD*, standard deviation; SUD, substance use disorder.

## References

[1] Kwon M, Park E, Dickerson SS (2019). Adolescent substance use and its association to sleep disturbances: A systematic review. Sleep Health.

[2] Koob GF, Colrain IM (2020). Alcohol use disorder and sleep disturbances: A feed-forward allostatic framework. Neuropsychopharmacology.

[3] Hasler BP, Smith LJ, Cousins JC, Bootzin RR (2012). Circadian rhythms, sleep, and substance abuse. Sleep Medicine Reviews.

[4] Meyrel M, Rolland B, Geoffroy PA (2020). Alterations in circadian rhythms following alcohol use: A systematic review. Prog Neuro-Psychopharmacol Biol Psychiatry.

[5] Hasler BP, Graves JL, Wallace ML (2022). Self-reported sleep and circadian characteristics predict alcohol and cannabis use: A longitudinal analysis of the National Consortium on Alcohol and Neurodevelopment in Adolescence Study. Alcohol Clin Exp Res.

[6] Troxel WM, Rodriguez A, Seelam R (2021). Longitudinal associations of sleep problems with alcohol and cannabis use from adolescence to emerging adulthood. Sleep.

[7] Miller MB, Donahue ML, Carey KB, Scott-Sheldon LA (2017). Insomnia treatment in the context of alcohol use disorder: A systematic review and meta-analysis. Drug Alcohol Depend.

[8] Duncan SC, Gau JM, Duncan TE, Strycker LA (2011). Development and correlates of alcohol use from ages 13–20. J Drug Educ.

[9] Czeisler CA, Gooley JJ (2007). Sleep and circadian rhythms in humans. Cold Spring Harb Symp Quant Biol.

[10] Crowley SJ, Van Reen E, LeBourgeois MK (2014). A longitudinal assessment of sleep timing, circadian phase, and phase angle of entrainment across human adolescence. PLoS One.

[11] Roenneberg T, Kuehnle T, Pramstaller PP (2004). A marker for the end of adolescence. Curr Biol.

[12] Gradisar M, Gardner G, Dohnt H (2011). Recent worldwide sleep patterns and problems during adolescence: A review and meta-analysis of age, region, and sleep. Sleep Med.

[13] Gariepy G, Danna S, Gobina I (2020). How are adolescents sleeping? Adolescent sleep patterns and sociodemographic differences in 24 European and North American countries. J Adolesc Health.

[14] Crowley SJ, Wolfson AR, Tarokh L, Carskadon MA (2018). An update on adolescent sleep: New evidence informing the perfect storm model. J Adolesc.

[15] Chang A-M, Aeschbach D, Duffy JF, Czeisler CA (2015). Evening use of light-emitting eReaders negatively affects sleep, circadian timing, and next-morning alertness. Proc Natl Acad Sci U S A.

[16] Bartel KA, Gradisar M, Williamson P (2015). Protective and risk factors for adolescent sleep: A meta-analytic review. Sleep Med Rev.

[17] Hirshkowitz M, Whiton K, Albert SM (2015). National Sleep Foundation’s sleep time duration recommendations: Methodology and results summary. Sleep Health.

[18] Roenneberg T, Pilz LK, Zerbini G, Winnebeck EC (2019). Chronotype and social jetlag: A (self-) critical review. Biology (Basel).

[19] Johnson EO, Roth T, Schultz L, Breslau N (2006). Epidemiology of DSM-IV insomnia in adolescence: Lifetime prevalence, chronicity, and an emergent gender difference. Pediatrics.

[20] Maslowsky J, Ozer EJ (2014). Developmental trends in sleep duration in adolescence and young adulthood: Evidence from a national United States sample. J Adolesc Health.

[21] Saelee R, Haardörfer R, Johnson DA, Gazmararian JA, Suglia SF (2023). Racial/ethnic and sex/gender differences in sleep duration trajectories from adolescence to adulthood in a US national sample. Am J Epidemiol.

[22] Buysse DJ (2014). Sleep health: Can we define it? Does it matter?. Sleep.

[23] Meltzer LJ, Williamson AA, Mindell JA (2021). Pediatric sleep health: It matters, and so does how we define it. Sleep Med Rev.

[24] Waddell JT, Jager J, Chassin L (2022). Maturing out of alcohol and cannabis co-use: A test of patterns and personality predictors. Alcohol Clin Exp Res.

[25] Maimaris W, McCambridge J (2014). Age of first drinking and adult alcohol problems: Systematic review of prospective cohort studies. J Epidemiol Community Health.

[26] Morean ME, Corbin WR, Fromme K (2012). Age of first use and delay to first intoxication in relation to trajectories of heavy drinking and alcohol-related problems during emerging adulthood. Alcohol Clin Exp Res.

[27] Morean ME, Kong G, Camenga DR, Cavallo DA, Connell C, Krishnan-Sarin S (2014). First drink to first drunk: Age of onset and delay to intoxication are associated with adolescent alcohol use and binge drinking. Alcohol Clin Exp Res.

[28] Stautz K, Cooper A (2013). Impulsivity-related personality traits and adolescent alcohol use: A meta-analytic review. Clin Psychol Rev.

[29] Lejuez CW, Magidson JF, Mitchell SH, Sinha R, Stevens MC, De Wit H (2010). Behavioral and biological indicators of impulsivity in the development of alcohol use, problems, and disorders. Alcohol Clin Exp Res.

[30] Magid V, MacLean MG, Colder CR (2007). Differentiating between sensation seeking and impulsivity through their mediated relations with alcohol use and problems. Addict Behav.

[31] MacPherson L, Magidson JF, Reynolds EK, Kahler CW, Lejuez C (2010). Changes in sensation seeking and risk-taking propensity predict increases in alcohol use among early adolescents. Alcohol Clin Exp Res.

[32] Littlefield AK, Stevens AK, Sher KJ (2014). Impulsivity and alcohol involvement: Multiple, distinct constructs and processes. Curr Addict Rep.

[33] Hittner JB, Swickert R (2006). Sensation seeking and alcohol use: A meta-analytic review. Addict Behav.

[34] Kang JI, Park CI, Sohn SY, Kim HW, Namkoong K, Kim SJ (2015). Circadian preference and trait impulsivity, sensation-seeking and response inhibition in healthy young adults. Chronobiol Int.

[35] Anderson C, Platten CR (2011). Sleep deprivation lowers inhibition and enhances impulsivity to negative stimuli. Behav Brain Research.

[36] Chan JKM, Trinder J, Andrewes HE, Colrain IM, Nicholas CL (2013). The acute effects of alcohol on sleep architecture in late adolescence. Alcohol Clin Exp Res.

[37] Chan JKM, Trinder J, Colrain IM, Nicholas CL (2015). The acute effects of alcohol on sleep electroencephalogram power spectra in late adolescence. Alcohol Clin Exp Res.

[38] Van Reen E, Arantes HB, Rupp TL, Maxwell JL, Acebo C, Carskadon MA (2006). Residual effects of a moderate dose of alcohol on sleep stages of young men. Sleep.

[39] Dijk D-J, Brunner DP, Aeschbach D, Tobler I, Borbély AA (1992). The effects of ethanol on human sleep EEG power spectra differ from those of benzodiazepine receptor agonists. Neuropsychopharmacology.

[40] Hu N, Ma Y, He J, Zhu L, Cao S (2020). Alcohol consumption and incidence of sleep disorder: A systematic review and meta-analysis of cohort studies. Drug Alcohol Depend.

[41] Fucito LM, Bold KW, Van Reen E (2018). Reciprocal variations in sleep and drinking over time among heavy-drinking young adults. J Abnorm Psychol.

[42] Miller MB, Freeman LK, Deroche CB, Park CJ, Hall NA, McCrae CS (2021). Sleep and alcohol use among young adult drinkers with insomnia: A daily process model. Addict Behav.

[43] Graupensperger S, Fairlie AM, Vitiello MV (2021). Daily-level effects of alcohol, marijuana, and simultaneous use on young adults’ perceived sleep health. Sleep.

[44] Goodhines PA, Gellis LA, Ansell EB, Park A (2019). Cannabis and alcohol use for sleep aid: A daily diary investigation. Health Psychol.

[45] Landolt H-P, Roth C, Dijk D-J, Borbély AA (1996). Late-afternoon ethanol intake affects nocturnal sleep and the sleep EEG in middle-aged men. J Clin Psychopharmacol.

[46] Brager AJ, Ruby CL, Prosser RA, Glass JD (2010). Chronic ethanol disrupts circadian photic entrainment and daily locomotor activity in the mouse. Alcohol Clin Exp Research.

[47] Brager AJ, Ruby CL, Prosser RA, Glass JD (2011). Acute ethanol disrupts photic and serotonergic circadian clock phase-resetting in the mouse. Alcohol Clin Exp Res.

[48] Burgess HJ, Rizvydeen M, Fogg LF, Keshavarzian A (2016). A single dose of alcohol does not meaningfully alter circadian phase advances and phase delays to light in humans. Am J Physiol Regul Integr Comp Physiol.

[49] Burgess HJ, Rizvydeen M, Kikyo F (2022). Sleep and circadian differences between light and heavy adult alcohol drinkers. Alcohol Clin Exp Res.

[50] Gonzalez D, Justin H, Reiss S (2022). Circadian rhythm shifts and alcohol access in adolescence synergistically increase alcohol preference and intake in adulthood in male C57BL/6 mice. Behav Brain Res.

[51] Graupensperger S, Fairlie AM, Ramirez JJ, Calhoun BH, Patrick ME, Lee CM (2022). Daily-level associations between sleep duration and next-day alcohol and cannabis craving and use in young adults. Addict Behav.

[52] Hasler BP, Casement MD, Sitnick SL, Shaw DS, Forbes EE (2017). Eveningness among late adolescent males predicts neural reactivity to reward and alcohol dependence 2 years later. Behav Brain Res.

[53] Hasler BP, Franzen PL, de Zambotti M (2017). Eveningness and later sleep timing are associated with greater risk for alcohol and marijuana use in adolescence: Initial findings from the National Consortium on Alcohol and Neurodevelopment in Adolescence Study. Alcohol Clin Exp Res.

[54] Hasler BP, Kirisci L, Clark DB (2016). Restless sleep and variable sleep timing during late childhood accelerate the onset of alcohol and other drug involvement. J Stud Alcohol Drugs.

[55] McGlinchey EL, Harvey AG (2015). Risk behaviors and negative health outcomes for adolescents with late bedtimes. J Youth Adolesc.

[56] Mike TB, Shaw DS, Forbes EE, Sitnick SL, Hasler BP (2016). The hazards of bad sleep—Sleep duration and quality as predictors of adolescent alcohol and cannabis use. Drug Alcohol Depend.

[57] Miller MB, Janssen T, Jackson KM (2017). The prospective association between sleep and initiation of substance use in young adolescents. J Adolesc Health.

[58] Nguyen-Louie TT, Brumback T, Worley MJ (2018). Effects of sleep on substance use in adolescents: A longitudinal perspective. Addict Biol.

[59] Pasch KE, Latimer LA, Cance JD, Moe SG, Lytle LA (2012). Longitudinal bi-directional relationships between sleep and youth substance use. J Youth Adolesc.

[60] Pieters S, Burk WJ, Van der Vorst H, Dahl RE, Wiers RW, Engels RCME (2015). Prospective relationships between sleep problems and substance use, internalizing and externalizing problems. J Youth Adolesc.

[61] Wong MM, Brower KJ, Fitzgerald HE, Zucker RA (2004). Sleep problems in early childhood and early onset of alcohol and other drug use in adolescence. Alcohol Clin Exp Res.

[62] Wong MM, Brower KJ, Nigg JT, Zucker RA (2010). Childhood sleep problems, response inhibition, and alcohol and drug outcomes in adolescence and young adulthood. Alcohol Clin Exp Res.

[63] Wong MM, Brower KJ, Zucker RA (2009). Childhood sleep problems, early onset of substance use and behavioral problems in adolescence. Sleep Med.

[64] Freeman D, Sheaves B, Waite F, Harvey AG, Harrison PJ (2020). Sleep disturbance and psychiatric disorders. Lancet Psychiatry.

[65] Zhang J, Lam SP, Li SX, Li AM, Lai KYC, Wing Y-K (2011). Longitudinal course and outcome of chronic insomnia in Hong Kong Chinese children: A 5-year follow-up study of a community-based cohort. Sleep.

[66] Warren CM, Riggs NR, Pentz MA (2017). Longitudinal relationships of sleep and inhibitory control deficits to early adolescent cigarette and alcohol use. J Adolesc.

[67] Haynie DL, Lewin D, Luk JW (2018). Beyond sleep duration: Bidirectional associations among chronotype, social jetlag, and drinking behaviors in a longitudinal sample of US high school students. Sleep.

[68] Troxel WM, Rodriguez A, Seelam R (2022). A latent class approach to understanding longitudinal sleep health and the association with alcohol and cannabis use during late adolescence and emerging adulthood. Addict Behav.

[69] Ho CY, Lin SH, Tsai MC, Yu T, Strong C (2020). Impact of cumulative unhealthy sleep practices in adolescence on substance use in young adulthood estimated using marginal structural modeling. Front Neurosci.

[70] Rezaie L, Fobian AD, McCall WV, Khazaie H (2018). Paradoxical insomnia and subjective-objective sleep discrepancy: A review. Sleep Med Rev.

[71] Hasler BP, Bruce S, Scharf D, Ngari W, Clark DB (2019). Circadian misalignment and weekend alcohol use in late adolescent drinkers: Preliminary evidence. Chronobiol Int.

[72] Stephenson M, Barr P, Ksinan A (2020). Which adolescent factors predict alcohol misuse in young adulthood? A co-twin comparisons study. Addiction.

[73] Hatoum AS, Winiger EA, Morrison CL, Johnson EC, Agrawal A (2022). Characterisation of the genetic relationship between the domains of sleep and circadian-related behaviours with substance use phenotypes. Addict Biol.

[74] Winiger EA, Ellingson JM, Morrison CL (2021). Sleep deficits and cannabis use behaviors: An analysis of shared genetics using linkage disequilibrium score regression and polygenic risk prediction. Sleep.

[75] Watson NF, Buchwald D, Harden KP (2013). A twin study of genetic influences on diurnal preference and risk for alcohol use outcomes. J Clin Sleep Med.

[76] Pasman JA, Smit DJ, Kingma L, Vink JM, Treur JL, Verweij KJH (2020). Causal relationships between substance use and insomnia. Drug Alcohol Depend.

[77] Vollmer C, Jankowski KS, Díaz-Morales JF, Itzek-Greulich H, Wüst-Ackermann P, Randler C (2017). Morningness–eveningness correlates with sleep time, quality, and hygiene in secondary school students: A multilevel analysis. Sleep Med.

[78] Tavernier R, Willoughby T (2014). Are all evening-types doomed? Latent class analyses of perceived morningness–eveningness, sleep and psychosocial functioning among emerging adults. Chronobiol Int.

[79] Kitamura S, Hida A, Watanabe M (2010). Evening preference is related to the incidence of depressive states independent of sleep-wake conditions. Chronobiol Int.

[80] Chan JWY, Lam SP, Li SX (2014). Eveningness and insomnia: Independent risk factors of nonremission in major depressive disorder. Sleep.

[81] Miller MB, DiBello AM, Carey KB, Borsari B, Pedersen ER (2017). Insomnia severity as a mediator of the association between mental health symptoms and alcohol use in young adult veterans. Drug Alcohol Depend.

[82] Kuerbis A, Padovano HT, Shao S, Houser J, Muench FJ, Morgenstern J (2018). Comparing daily drivers of problem drinking among older and younger adults: An electronic daily diary study using smartphones. Drug Alcohol Depend.

[83] Creswell KG (2021). Drinking together and drinking alone: A social-contextual framework for examining risk for alcohol use disorder. Curr Dir Psychol Sci.

[84] Moos RH, Moos BS (2006). Rates and predictors of relapse after natural and treated remission from alcohol use disorders. Addiction.

[85] Fucito LM, DeMartini KS, Hanrahan TH, Yaggi HK, Heffern C, Redeker NS (2017). Using sleep interventions to engage and treat heavy-drinking college students: A randomized pilot study. Alcohol Clin Exp Res.

[86] Fucito LM, DeMartini KS, Hanrahan TH, Whittemore R, Yaggi HK, Redeker NS (2015). Perceptions of heavy-drinking college students about a sleep and alcohol health intervention. Behav Sleep Med.

[87] Fucito LM, Ash GI, DeMartini KS (2021). A multimodal mobile sleep intervention for young adults engaged in risky drinking: Protocol for a randomized controlled trial. JMIR Research Protocols.

[88] Miller MB, Deroche CB, Freeman LK (2021). Cognitive behavioral therapy for insomnia among young adults who are actively drinking: A randomized pilot trial. Sleep.

[89] Miller MB, Freeman L, Park CJ (2021). Insomnia treatment effects among young adult drinkers: Secondary outcomes of a randomized pilot trial. Alcohol Clin Exp Res.

[90] Hasler BP, Clark DB (2013). Circadian misalignment, reward-related brain function, and adolescent alcohol involvement. Alcohol Clin Exp Res.

[91] Hasler BP, Pedersen SL (2020). Sleep and circadian risk factors for alcohol problems: A brief overview and proposed mechanisms. Curr Opin Psychol.

[92] Logan RW, Hasler BP, Forbes EE (2018). Impact of sleep and circadian rhythms on addiction vulnerability in adolescents. Biol Psychiatry.

[93] Swartz JR, Weissman DG, Ferrer E (2020). Reward-related brain activity prospectively predicts increases in alcohol use in adolescents. J Am Acad Child Adolesc Psychiatry.

[94] Morales AM, Jones SA, Ehlers A, Lavine JB, Nagel BJ (2018). Ventral striatal response during decision making involving risk and reward is associated with future binge drinking in adolescents. Neuropsychopharmacology.

[95] Venkatraman V, Chuah YML, Huettel SA, Chee MWL (2007). Sleep deprivation elevates expectation of gains and attenuates response to losses following risky decisions. Sleep.

[96] Mullin BC, Phillips ML, Siegle GJ, Buysse DJ, Forbes EE, Franzen PL (2013). Sleep deprivation amplifies striatal activation to monetary reward. Psychol Med.

[97] Gujar N, Yoo SS, Hu P, Walker MP (2011). Sleep deprivation amplifies reactivity of brain reward networks, biasing the appraisal of positive emotional experiences. J Neurosci.

[98] Hasler BP, Soehner AM, Wallace ML (2021). Experimentally imposed circadian misalignment alters the neural response to monetary rewards and response inhibition in healthy adolescents. Psychol Med.

[99] Hasler BP, Sitnick SL, Shaw DS, Forbes EE (2013). An altered neural response to reward may contribute to alcohol problems among late adolescents with an evening chronotype. Psychiatry Res.

[100] Hasler BP, Graves JL, Soehner AM, Wallace ML, Clark DB (2022). Preliminary evidence that circadian alignment predicts neural response to monetary reward in late adolescent drinkers. Front Neurosci.

[101] Treloar H, Celio MA, Lisman SA, Miranda R, Spear LP (2017). Subjective alcohol responses in a cross-sectional, field-based study of adolescents and young adults: Effects of age, drinking level, and dependence/consequences. Drug Alcohol Depend.

[102] Spear LP (2018). Effects of adolescent alcohol consumption on the brain and behaviour. Nat Rev Neurosci.

[103] Miranda R, Monti PM, Ray L (2014). Characterizing subjective responses to alcohol among adolescent problem drinkers. J Abnorm Psychol.

[104] Chavarria J, Fridberg DJ, Obst E, Zimmermann US, King AC (2021). Subjective alcohol responses in high- and low-risk adolescents: Results from the Dresden Longitudinal Study on Alcohol Use in Young Adults. Addiction.

[105] King A, Vena A, Hasin DS, de Wit H, O’Connor SJ, Cao D (2021). Subjective responses to alcohol in the development and maintenance of alcohol use disorder. Am J Psychiatry.

[106] Van Reen E, Rupp TL, Acebo C, Seifer R, Carskadon MA (2013). Biphasic effects of alcohol as a function of circadian phase. Sleep.

[107] Hasler BP, Wallace ML, White SJ, Molina BS, Pedersen SL (2019). Preliminary evidence that real world sleep timing and duration are associated with laboratory-assessed alcohol response. Alcohol Clin Exp Res.

[108] Grant VV, Stewart SH, O’Connor RM, Blackwell E, Conrod PJ (2007). Psychometric evaluation of the five-factor Modified Drinking Motives Questionnaire—Revised in undergraduates. Addict Behav.

[109] Digdon N, Landry K (2013). University students’ motives for drinking alcohol are related to evening preference, poor sleep, and ways of coping with stress. Biol Rhythm Res.

[110] Chapman DP, Wheaton AG, Anda RF (2011). Adverse childhood experiences and sleep disturbances in adults. Sleep Med.

[111] Johnson V, Pandina RJ (1993). A longitudinal examination of the relationships among stress, coping strategies, and problems associated with alcohol use. Alcohol Clin Exp Res.

[112] Yap Y, Slavish DC, Taylor DJ, Bei B, Wiley JF (2020). Bi-directional relations between stress and self-reported and actigraphy-assessed sleep: A daily intensive longitudinal study. Sleep.

[113] Ayer LA, Harder VS, Rose GL, Helzer JE (2011). Drinking and stress: An examination of sex and stressor differences using IVR-based daily data. Drug Alcohol Depend.

[114] Kalmbach DA, Anderson JR, Drake CL (2018). The impact of stress on sleep: Pathogenic sleep reactivity as a vulnerability to insomnia and circadian disorders. J Sleep Res.

[115] Russell MA, Almeida DM, Maggs JL (2017). Stressor-related drinking and future alcohol problems among university students. Psychol Addict Behav.

[116] Lund HG, Reider BD, Whiting AB, Prichard JR (2010). Sleep patterns and predictors of disturbed sleep in a large population of college students. J Adolesc Health.

[117] Taylor DJ, Bramoweth AD (2010). Patterns and consequences of inadequate sleep in college students: Substance use and motor vehicle accidents. J Adolesc Health.

[118] Terry-McElrath YM, O’Malley PM, Johnston LD (2009). Reasons for drug use among American youth by consumption level, gender, and race/ethnicity: 1976–2005. J Drug Issues.

[119] Hasler BP, Soehner AM, Clark DB (2015). Sleep and circadian contributions to adolescent alcohol use disorder. Alcohol.

[120] Roehrs T, Roth T (2018). Insomnia as a path to alcoholism: Tolerance development and dose escalation. Sleep.

[121] Roehrs T, Papineau K, Rosenthal L, Roth T (1999). Ethanol as a hypnotic in insomniacs: Self administration and effects on sleep and mood. Neuropsychopharmacology.

[122] Miller MB, Curtis AF, Hall NA (2022). Daily associations between modifiable sleep behaviors and nighttime sleep among young adult drinkers with insomnia. J Clin Sleep Med.

[123] Hug E, Winzeler K, Pfaltz MC, Cajochen C, Bader K (2019). Later chronotype is associated with higher alcohol consumption and more adverse childhood experiences in young healthy women. Clocks & Sleep.

[124] Lowman C, Hunt WA, Litten RZ, Drummond DC (2000). Research perspectives on alcohol craving: An overview. Addiction.

[125] Hisler GC, Pedersen SL, Hasler BP (2022). The 24-hour rhythm in alcohol craving and individual differences in sleep characteristics and alcohol use frequency. Alcohol Clin Exp Res.

[126] Hisler GC, Rothenberger SD, Clark DB, Hasler BP (2021). Is there a 24-hour rhythm in alcohol craving and does it vary by sleep/circadian timing?. Chronobiol Int.

[127] Baskerville W-A, Grodin EN, Venegas A, Ray LA (2022). Global sleep quality is associated with tonic craving, but not cue-induced craving. Addict Behav.

[128] James MH, Mahler SV, Moorman DE, Aston-Jones G (2017). A decade of orexin/hypocretin and addiction: Where are we now?. Curr Top Behav Neurosci.

[129] Hopf FW (2020). Recent perspectives on orexin/hypocretin promotion of addiction-related behaviors. Neuropharmacology.

[130] ClinicalTrials.gov [Internet].

[131] ClinicalTrials.gov [Internet].

[132] Settles RF, Cyders M, Smith GT (2010). Longitudinal validation of the acquired preparedness model of drinking risk. Psychol Addict Behav.

[133] Bauducco SV, Salihovic S, Boersma K (2019). Bidirectional associations between adolescents’ sleep problems and impulsive behavior over time. Sleep Med X.

[134] Hasler BP, Wallace ML, Graves JL, Molina BSG, Pedersen SL (2022). Circadian preference is associated with multiple domains of trait and state level impulsivity. Chronobiol Int.

[135] Grant BF, Chou SP, Saha TD (2017). Prevalence of 12-month alcohol use, high-risk drinking, and DSM-IV alcohol use disorder in the United States, 2001–2002 to 2012–2013: Results From the National Epidemiologic Survey on Alcohol and Related Conditions. JAMA Psychiatry.

[136] White A, Castle IJ, Chen CM, Shirley M, Roach D, Hingson R (2015). Converging patterns of alcohol use and related outcomes among females and males in the United States, 2002 to 2012. Alcohol Clin Exp Res.

[137] White AM (2020). Gender differences in the epidemiology of alcohol use and related harms in the United States. Alcohol Res.

[138] Zhang J, Chan NY, Lam SP (2016). Emergence of sex differences in insomnia symptoms in adolescents: A large-scale school-based study. Sleep.

[139] Roenneberg T, Kuehnle T, Juda M (2007). Epidemiology of the human circadian clock. Sleep Med Rev.

[140] Rognmo K, Bergvik S, Rosenvinge JH, Bratlid KL, Friborg O (2019). Gender differences in the bidirectional relationship between alcohol consumption and sleeplessness: The Tromsø study. BMC Public Health.

[141] Arnedt JT, Rohsenow DJ, Almeida AB (2011). Sleep following alcohol intoxication in healthy, young adults: Effects of sex and family history of alcoholism. Alcohol Clin Exp Res.

[142] Koob GF (2009). Brain stress systems in the amygdala and addiction. Brain Res.

[143] Peltier MR, Verplaetse TL, Mineur YS (2019). Sex differences in stress-related alcohol use. Neurobiol Stress.

[144] Verplaetse TL, Moore KE, Pittman BP (2018). Intersection of stress and gender in association with transitions in past year DSM-5 substance use disorder diagnoses in the United States. Chronic Stress (Thousand Oaks).

[145] Bryant BE, Jordan A, Clark US (2022). Race as a social construct in psychiatry research and practice. JAMA Psychiatry.

[146] Belloir JA, Kidd JD, Dworkin JD, Bockting WO (2022). Examining the role of problematic drug use in the relationship between discrimination and sleep disturbance in transgender and nonbinary individuals. Addict Behav.

[147] Butler ES, McGlinchey E, Juster RP (2020). Sexual and gender minority sleep: A narrative review and suggestions for future research. J Sleep Res.

[148] Gilbert PA, Pass LE, Keuroghlian AS, Greenfield TK, Reisner SL (2018). Alcohol research with transgender populations: A systematic review and recommendations to strengthen future studies. Drug Alcohol Depend.

[149] Harry-Hernandez S, Reisner SL, Schrimshaw EW (2019). 0692 Gender dysphoria, mental health, and poor sleep quality among transgender and gender non-conforming individuals: A qualitative study in New York City. Sleep.

[150] Levenson JC, Thoma BC, Hamilton JL, Choukas-Bradley S, Salk RH (2021). Sleep among gender minority adolescents. Sleep.

[151] Patterson CJ, Potter EC (2019). Sexual orientation and sleep difficulties: A review of research. Sleep Health.

[152] Caceres BA, Hickey KT (2020). Examining sleep duration and sleep health among sexual minority and heterosexual adults: Findings from NHANES (2005–2014). Behav Sleep Med.

[153] Meyer IH (2003). Prejudice, social stress, and mental health in lesbian, gay, and bisexual populations: Conceptual issues and research evidence. Psychol Bull.

[154] Hatzenbuehler ML, Nolen-Hoeksema S, Dovidio J (2009). How does stigma “get under the skin”?: The mediating role of emotion regulation. Psychol Sci.

[155] Hequembourg AL, Brallier SA (2009). An exploration of sexual minority stress across the lines of gender and sexual identity. J Homosex.

[156] Kalb N, Gillis JR, Goldstein AL (2018). Drinking to cope with sexual minority stressors: Understanding alcohol use and consequences among LGBQ emerging adults. J Gay Lesbian Mental Health.

[157] Johnson DA, Reiss B, Cheng P, Jackson CL (2022). Understanding the role of structural racism in sleep disparities: A call to action and methodological considerations. Sleep.

[158] Jackson CL, Walker JR, Brown MK, Das R, Jones NL (2020). A workshop report on the causes and consequences of sleep health disparities. Sleep.

[159] Shield KD, Gmel G, Kehoe-Chan T, Dawson DA, Grant BF, Rehm J (2013). Mortality and potential years of life lost attributable to alcohol consumption by race and sex in the United States in 2005. PLoS One.

[160] Mulia N, Ye Y, Greenfield TK, Zemore SE (2009). Disparities in alcohol-related problems among White, Black, and Hispanic Americans. Alcohol Clin Exp Res.

[161] Herd D (1994). Predicting drinking problems among black and white men: Results from a national survey. J Stud Alcohol.

[162] Grant BF, Chou SP, Saha TD (2017). Prevalence of 12-month alcohol use, high-risk drinking, and DSM-IV alcohol use disorder in the United States, 2001–2002 to 2012–2013: Results from the National Epidemiologic Survey on Alcohol and Related Conditions. JAMA Psychiatry.

[163] Grandner MA, Williams NJ, Knutson KL, Roberts D, Jean-Louis G (2016). Sleep disparity, race/ethnicity, and socioeconomic position. Sleep Med.

[164] Fuller-Rowell TE, Curtis DS, El-Sheikh M, Chae DH, Boylan JM, Ryff CD (2016). Racial disparities in sleep: The role of neighborhood disadvantage. Sleep Med.

[165] Edwards S, Reeves GM, Fishbein D (2015). Integrative model of the relationship between sleep problems and risk for youth substance use. Curr Addict Rep.

[166] Irwin M, Miller C, Gillin JC, Demodena A, Ehlers CL (2000). Polysomnographic and spectral sleep EEG in primary alcoholics: An interaction between alcohol dependence and African-American ethnicity. Alcohol Clin Exp Res.

[167] Jackson CL, Gaston SA, Liu R, Mukamal K, Rimm EB (2018). The relationship between alcohol drinking patterns and sleep duration among black and white men and women in the United States. Int J Environ Res Public Health.

[168] Ehlers CL, Wills D, Gilder DA (2018). A history of binge drinking during adolescence is associated with poorer sleep quality in young adult Mexican Americans and American Indians. Psychopharmacology (Berl).

[169] Boness CL, Hasler BP, Sheehan H, Pedersen SL (2022). Associations between specific sleep and circadian characteristics and alcohol use disorder criteria and problems. Addict Behav.

[170] Hasler BP, Martin CS, Wood DS, Rosario B, Clark DB (2014). A longitudinal study of insomnia and other sleep complaints in adolescents with and without alcohol use disorders. Alcohol Clin Exp Res.

[171] Miller MB, DiBello AM, Lust SA, Carey MP, Carey KB (2016). Adequate sleep moderates the prospective association between alcohol use and consequences. Addict Beh.

[172] Wong MM, Robertson GC, Dyson RB (2015). Prospective relationship between poor sleep and substance-related problems in a national sample of adolescents. Alcohol Clin Exp Res.

